# *Prevotella* species in the human gut is primarily comprised of *Prevotella copri*, *Prevotella stercorea* and related lineages

**DOI:** 10.1038/s41598-022-12721-4

**Published:** 2022-05-31

**Authors:** Yun Kit Yeoh, Yang Sun, Lawrence Yuk Ting Ip, Lan Wang, Francis K. L. Chan, Yinglei Miao, Siew C. Ng

**Affiliations:** 1 Microbiota I-Center, Shatin, Hong Kong SAR China; 2 Centre for Gut Microbiota Research, Faculty of Medicine, The Chinese University of Hong Kong, Shatin, Hong Kong SAR China; 3 Department of Microbiology, Faculty of Medicine, The Chinese University of Hong Kong, Shatin, Hong Kong SAR China; 4 Department of Gastroenterology, The First Affiliated Hospital of Kunming Medical University, Kunming, Yunnan China; 5 Yunnan Province Clinical Research Centre for Digestive Diseases, Kunming, Yunnan China; 6 Department of Medicine and Therapeutics, Institute of Digestive Disease, Li Ka Shing Institute of Health Sciences, Faculty of Medicine, The Chinese University of Hong Kong, Shatin, Hong Kong SAR China

**Keywords:** Microbial ecology, Microbiome

## Abstract

*Prevotella* species in the human gut microbiome are primarily comprised of *Prevotella copri*, and its diversity and function were recently investigated in detail. Much less is known about other *Prevotella* species in the human gut. Here, we examined the composition of *Prevotella* species in human guts by mapping publicly available gut metagenomes to a dereplicated set of metagenome-assembled genomes (MAGs) representing *Prevotella* lineages found in human guts. In most human cohorts, *P. copri* is the most relatively abundant species (e.g. up to 14.3% relative abundance in Tangshan, China). However, more than half of the metagenome reads in several cohorts mapped to *Prevotella* MAGs representing *P. stercorea* and several other species sister to *P. stercorea* and *P. copri*. Analyses of genes encoded in these genomes indicated that *P. stercorea* and related lineages lacked many hemicellulose degrading enzymes and were thus less likely to metabolise hemicelluloses compared with *P. copri* and copri-related lineages. Instead, *P. stercorea* genomes possess several carbohydrate esterases that may be involved in releasing ester modifications from carbohydrates to facilitate their degradation. These findings reveal unexplored *Prevotella* diversity in the human gut and indicate possible niche partitions among these related species.

## Introduction

Several *Prevotella* species inhabit the human gut, among which *Prevotella copri* is the most common with estimates indicating ~ 40% prevalence in the wider human population and relative abundances that exceed 50% in some individuals^1^. This species is more prevalent in non-Western populations likely due to its association with high fibre low fat diets^2–4^, and is often linked with desirable health measures such as reduced visceral fat and improved glucose metabolism^5,6^. However, there are conflicting reports that implicate *P. copri* in adverse conditions such as insulin resistance^7^, hypertension^8^ and persistent gut inflammation^9^. Recently, Tett and colleagues demonstrated that the existing *P. copri* lineage is comprised of at least four distinct species-level lineages each sharing less than 95% average nucleotide identity with one another^1^. Furthermore, the use of polysaccharides among *P. copri* isolates can markedly vary indicating distinct metabolic patterns within this lineage^10^. These inconsistencies between traditional taxonomic designations and species boundaries coupled with strain-specific metabolic diversity and dietary preferences could be reasons behind the conflicting observations related to *P. copri* and human health^11^, and more broadly underscores the need to characterise microbial diversity in the human microbiome to understand its function.

Other than *P. copri*, the human gut hosts several other *Prevotella* species such as *P. stercorea*^12^, *P. rectalis*^13^, *P. histicola*^14^ and some only known by genomes recovered from human gut metagenomes^15–18^. Of these, *P. stercorea* is likely the second most prevalent and relatively abundant species in the healthy human gut after *P. copri* based on healthy human gut metagenomes^19^ while much less is known about the distribution of other *Prevotella* species. For example, *P. rectalis*^13^ and *P. ihumii*^20^ were recently isolated from individuals with no apparent disease and to our knowledge have not been implicated in any health conditions. Furthermore, DNA-based gut microbiome analyses often mention unclassified *Prevotella* but fall short of pinpointing exact species^21,22^ likely due to uncharacterised diversity in this lineage.

Since *P. copri* are important members of the human gut microbiome due to their roles in human health as well as putative associations with several adverse conditions, we hypothesised that uncharacterised *Prevotella* species could also play important functions in the human gut as they are likely to share similar traits with *P. copri*. As such, an assessment of overall *Prevotella* diversity and distribution in the human gut will greatly facilitate understanding of their roles in health. Here, we examined the genomes of *Prevotella* species recovered from human gut metagenomes and show that the most prevalent and relatively abundant species are primarily related to *P. copri* and *P. stercorea*, and these species possess distinct gene repertoires likely reflecting adaptations to metabolic niches. Specifically, *P. stercorea* and related species lack several genes for xylan metabolism but have carbohydrate esterases that suggest they work cooperatively with *P. copri* in metabolising dietary fibre.

## Methods

### Obtaining *Prevotella* genomes from public repositories and gut metagenome data sets

This study was approved by the Joint Chinese University of Hong Kong New Territories East Cluster Clinical Research Ethics Committee (reference number 2016.707 and 2016.407) and was performed in accordance with relevant guidelines, regulations, and the Declaration of Helsinki. Written informed consent was obtained from all participants. Microbial genomes were downloaded from publicly available sources including the Genome Taxonomy Database (GTDB)^23^ R202 which contains RefSeq genomes and from several studies of human microbiome diversity^15,17,18^. In addition, we obtained *Prevotella* genomes from two previous studies examining gut microbiome diversity in Hong Kong^24^ and Yunnan^25^. For these two studies, metagenome sequences were quality filtered using Trimmomatic^26^ v0.39 to remove adapter sequences and low-quality regions. Quality filtered reads were then de novo assembled using MEGAHIT^27^ v1.2.9. Metagenome-assembled genomes (MAGs) were reconstructed from each metagenome assembly by mapping quality filtered reads to their respective assemblies using BWA MEM^28^ v0.7.17 and then using these coverage profiles as input into MetaBAT^29^ v2.10.2, v2.12.1 and MaxBin^30^ v2.2.7. The resulting MAGs were dereplicated using DAS Tool^31^ and checked for completeness and contamination using CheckM^32^ (Parks et al., 2015) v1.1.3. Taxonomy was inferred using GTDB-Tk^33^ v1.4.1 with the R95 database.

### Inferring *Prevotella* phylogeny

In addition to completeness and contamination estimates from CheckM and taxonomies from GTDB-Tk, all MAGs were scanned using Kraken2^34^ v2.1.1 with the StandardPlusPF precompiled database (v20210517) to assess the fraction of each genome consistent with taxonomy inferred from GTDB-Tk to avoid including chimeric genome assemblies. Genomes that scored > 90% completeness and < 5% contamination based on CheckM, inferred as *Prevotella* by GTDB-Tk and had > 70% of their genomes classified as *Prevotella* by Kraken2 were selected for phylogenetic inference and genome annotation. Identical genomes were dereplicated based on a threshold of 99% average nucleotide identity (ANI) using CoverM v0.6.0 (https://github.com/wwood/CoverM). The genomes were also dereplicated at 95% ANI to obtain a set of unique species. Bootstrapped phylogenetic tree (1000 bootstraps) was inferred using IQ-TREE2^35^ v2.1.2 based on a concatenated alignment of 120 phylogenetically informative single copy bacterial marker genes generated by GTDB-Tk for both the set of 99% and 95% ANI genomes.

### Estimating *Prevotella* prevalence and relative abundance in diverse human populations

To estimate the prevalence and abundance of *Prevotella* in the human population, we downloaded publicly available human gut metagenomes (Table S1) from countries including Austria^36^, Denmark, Spain^37^, France^38^, Sweden^39^, China^8,40^, Japan^41^, El Salvador, Peru^42^, Fiji^43^, Israel^44^, Mongolia^45^, Tanzania^46,47^ and USA^48,49^. These data were quality filtered using Trimmomatic v0.39 and then mapped to the species dereplicated set of *Prevotella* genomes (95% ANI) using CoverM v0.6.0 which produces relative abundance estimates of each genome in each cohort based on coverage and number of mapped reads (see https://github.com/wwood/CoverM/#calculation-methods). Prevalence was calculated as the number of metagenomes in which *Prevotella* was detected divided by the total number of metagenomes in each cohort. For surveys with case–control cohorts (e.g. colorectal cancer in Japan^41^), only non-disease subjects were included in this study.

### Annotating genes

We used Panaroo^50^ v1.2.8 to determine gene orthologues shared among the *Prevotella* genomes. Orthologues were assigned Kyoto Encyclopedia of Genes and Genomes (KEGG) Orthologs (KOs) and Carbohydrate active enzymes (CAZy) families by searching against a database of Hidden Markov Models implemented in EnrichM (https://github.com/geronimp/enrichM) v0.5.0. Distribution of KOs and CAZy families among the MAGs were visualised using non-metric multidimensional scaling (NMDS) ordinations on Jaccard distances using the Vegan package^51^ v2.5.7 in R v4.0.3.

## Results

### Phylogenetic diversity of human gut associated *Prevotella* species

To assess the extent of *Prevotella* diversity in the human gut, we first downloaded publicly available collections of MAGs derived from human gut metagenomes^15,17,18^. Additionally, we obtained MAGs from gut metagenome surveys of Hong Kong^24^ and Yunnan populations^25^ as we were interested in the distribution of *Prevotella* in these locales. From these data sets, we selected MAGs classified as members of the *Prevotella* genus based on GTDB taxonomy. After quality filtering and dereplication at 99% ANI to remove identical genomes, 3943 MAGs remained of which more than 60% were comprised of five highly represented species including *P. copri* (1579 genomes), *P. sp003447235* (416 genomes), *P. sp000434975* (185 genomes), *P. stercorea* (174 genomes) and *P. sp002265625* (164 genomes) (Tables S2 and Table S3).

**Figure 1:**
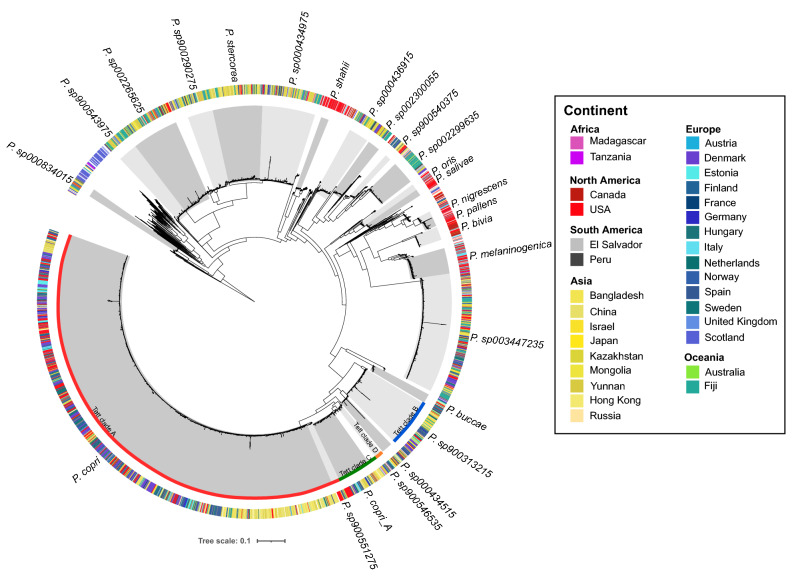
Phylogenetic tree representing phylogenetic relationships among *Prevotella* isolates and metagenome-assembled genomes (MAGs) recovered from human gut metagenomes. Five *Paraprevotella* genomes were used as outgroup to root the tree. MAGs included in this tree were downloaded from publicly available repositories^15,17,18^ and from healthy human gut metagenome surveys of Hong Kong^24^ and Yunnan populations^25^. Genomes were dereplicated at 99% average nucleotide identity (ANI) and a concatenated amino acid alignment consisting of 120 phylogenetically informative marker genes (from GTDB) was used to infer phylogenetic trees using IQTREE2. Taxonomy labels from GTDB are shown for species represented by at least 20 genomes (i.e., lower odds of spurious misassemblies or genome binning) (see Table S2 for genome counts); species boundaries are indicated by the alternating grey shades. Note that the GTDB-based taxonomy used here is not comparable to the four *P. copri* clades A–D from Tett et al.^1^- these four clades from Tett et al.^1^ are indicated separately in the figure. The outer colour strip indicates source country/continent of the respective genomes shaded by continent.

To establish phylogenetic relationships among the 3943 MAGs representing *Prevotella* species detected in the guts of the wider human population, we downloaded an additional 222 NCBI RefSeq *Prevotella* genomes held in the GTDB database and incorporated these with the set of 3943 MAGs. Phylogenetic trees were then inferred based on a concatenated alignment of 120 bacterial single copy marker genes to produce a bootstrapped consensus tree (Fig. 1). Within the lineage consisting of *P. copri* species, MAGs from the Asian continent were phylogenetically closer to one another compared with those from Western geographies (USA, Canada, Europe) possibly reflecting adaptations to local populations. These patterns were also observed in *P. stercorea* in which 128 out of 174 genomes were derived from Asian populations. Conversely, species such as *P. pallens* (24 of 33), *P. salivae* (18 of 23)*,* and *P. bivia* (41 of 47) were largely represented by MAGs from Canada and USA. As for MAGs derived from gut metagenomes representing rural populations (e.g. Tanzania, Fiji, El Salvador), they were primarily classified as several not yet formally characterised species including sp002299635, sp002265625, sp000434975 and sp900543975. These observations indicate that numerous *Prevotella* species are found in human guts (at least 25 when considering those represented by > 20 MAGs for confidence; Table S2), many of which have not been formally characterised especially those from rural populations.

### Uncharacterised *Prevotella* species are prevalent and relatively abundant in the human gut

**Figure 2:**
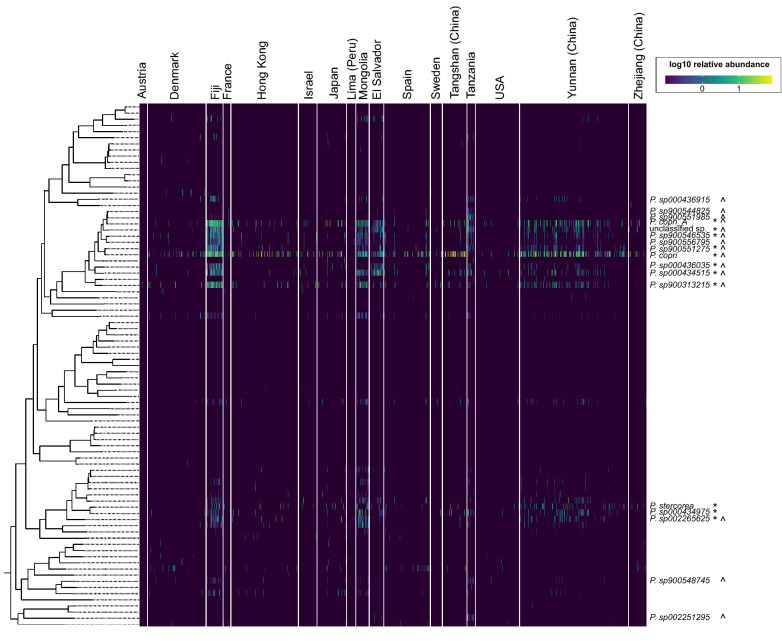
Estimated *Prevotella* relative abundances across several human gut metagenome data sets. Phylogenetic tree indicates relationships of the *Prevotella* genomes dereplicated at the species level (> 95% average nucleotide identity). Only genomes with reads mapped are included in this figure. Taxonomy labels are shown for species with at least 1% relative abundance (indicated with *) or at least 50% prevalence (indicated with ^) in any one data set. See Table S4 for relative abundance and prevalence values.

Since many of these *Prevotella* MAGs are directly derived from human gut metagenomes and may not be identified using conventional microbial community profiling tools, we sought to estimate their prevalence and relative abundances in the human population by mapping metagenome reads. In total, we downloaded 4095 human gut metagenome data sets from non-disease cohorts representing populations from China, Japan, USA, Austria, Denmark, France, Spain, Israel, Sweden, El Salvador, Peru, Fiji, Mongolia, and Tanzania, and mapped quality filtered reads to the set of *Prevotella* MAGs dereplicated at the species level (95% ANI). As expected, total *Prevotella* relative abundance was highest in Fiji, a rural population data set (average 36.1% relative abundance), followed by Tangshan (China; 20.5%) and Mongolia (19.6%) (Fig. 2) (Table 1). They were also more widespread in rural communities such as Fiji (97.8% prevalence), El Salvador (94.7%) and Mongolia (91.8%) compared with urban cohorts in which prevalence ranged between 11 and 58% (Table 1). Among the *Prevotella* species identified above, *P. copri* was the most prevalent (average 30.7%) and relatively abundant overall (average 3.3%) (Table 2, Table S4). Other prevalent and relatively abundant species were primarily from two distinct lineages, one more closely related to *P. copri* and the second to *P. stercorea* (hereafter referred to as copri and stercorea groups) (Fig. 2) (Table S4). Collectively, these species other than *P. copri* account for an average 4.3% relative abundance which is comparable to the 3.2% for *P. copri* (Table S5). Like *P. copri*, most of them are more prevalent and relatively abundant in rural populations such as Fiji, Mongolia and El Salvador, possibly indicating a similar association with non-Western lifestyles and/or high fibre diets. In addition, we detected a few lineages at lower prevalence and relative abundances that are possibly more restricted in their distribution such as *P. sp001275135* and *P. sp003447235* (Table S4). For example, *P. sp001275135* has an average prevalence of only 3.7% across all data sets included in this study, but its prevalence in France and Spain is 14.8% and 17.5%, respectively. Taken together, these observations indicate that the most common *Prevotella* species in the human gut are primarily comprised of *P. copri*, *P. stercorea* and several closely related lineages, although some species with more limited distributions may be prevalent in certain biogeographies.

**Table 1:** Distribution of total Prevotella in the human gut.

Data set	Relative abundance ± standard deviation (%)	Prevalence (%)	Number of metagenomes
Austria	0.09 ± 0.35	11.11	63
Denmark	2.42 ± 6.19	41.35	474
Fiji	36.08 ± 17.64	97.83	138
France	2.26 ± 5.99	52.46	61
Hong Kong	6.16 ± 13.21	33.27	547
Israel	5.82 ± 10.34	58.00	150
Japan	8.15 ± 14.75	41.95	236
Lima (Peru)	0.37 ± 1.56	19.48	77
Mongolia	19.61 ± 16.05	91.82	110
El Salvador	10.24 ± 9.70	94.74	114
Spain	3.38 ± 9.69	39.68	378
Sweden	2.58 ± 9.87	37.00	100
Tangshan	20.48 ± 24.84	56.63	196
Tanzania	7.36 ± 8.85	79.10	67
USA	1.12 ± 4.85	17.60	358
Yunnan rural	8.22 ± 12.32	69.32	427
Yunnan urban	8.70 ± 13.67	56.39	454
Zhejiang	4.87 ± 10.66	37.93	145

**Table 2:** Most relatively abundant and prevalent *Prevotella* species in the human gut.

Species (GTDB taxonomy)	Average relative abundance ± standard deviation (%)	Average prevalence (%)
*Prevotella copri*	3.27 ± 3.62	30.73 ± 26.43
*Prevotella copri_ A*	1.10 ± 1.59	20.11 ± 25.05
*Prevotella sp000434515*	0.59 ± 0.82	16.89 ± 25.17
*Prevotella sp900313215*	0.58 ± 0.92	14.44 ± 20.81
*Unclassified Prevotella*	0.37 ± 0.61	15.53 ± 25.16
*Prevotella sp000434975*	0.28 ± 0.43	10.22 ± 14.77
*Prevotella stercorea*	0.25 ± 0.29	8.59 ± 8.51
*Prevotella sp900546535*	0.25 ± 0.36	14.18 ± 22.96
*Prevotella sp002265625*	0.20 ± 0.38	10.48 ± 18.11
*Prevotella sp900551275*	0.19 ± 0.36	14.47 ± 23.20
*Prevotella sp000436035*	0.16 ± 0.37	8.05 ± 16.36
*Prevotella sp900290275*	0.11 ± 0.16	7.31 ± 13.58
*Prevotella sp900556795*	0.10 ± 0.21	14.75 ± 25.02
*Prevotella sp000436915*	0.09 ± 0.15	8.83 ± 16.88
*Prevotella sp900543975*	0.09 ± 0.21	7.43 ± 15.23

### Human gut *Prevotella* species occupy distinct functional niches

**Figure 3:**
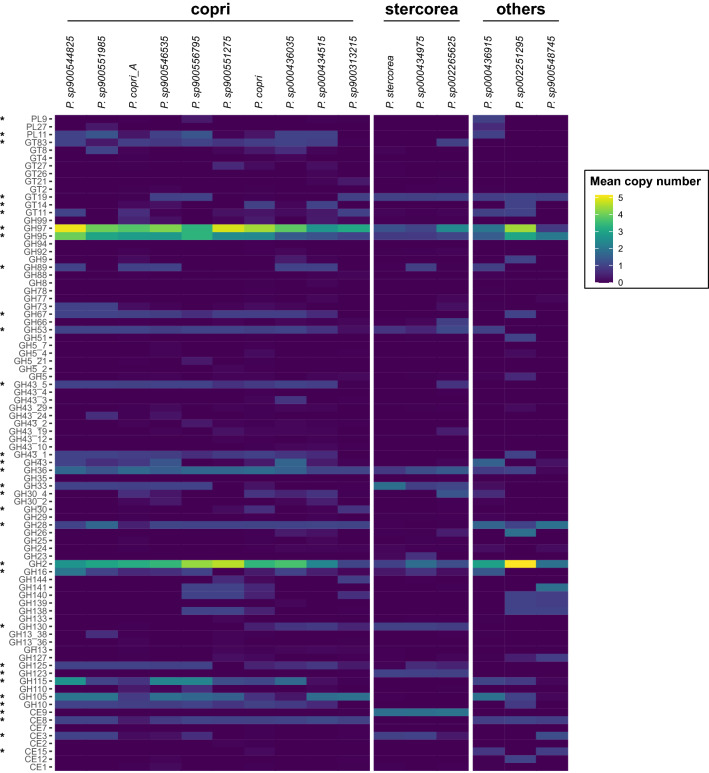
Distribution of carbohydrate active enzyme (CAZy) families in human gut *Prevotella* (average copies per genome). Only genomes with > 1% relative abundance or > 50% prevalence as indicated in Fig. 2 are included. CAZy families were identified in the genomes using Hidden Markov Models. Asterisks indicate families with statistically different copy counts (FDR < 0.001 Kruskal Wallis test with false discovery rate adjustment) and with at least one copy in any one of the three groups (copri, stercorea or other). See Table S7 for copy numbers. *CE* carbohydrate esterase, *GH* glycoside hydrolase, *GT* glycosyl transferase, *PL* polysaccharide lyase.

*Prevotella copri* in the human gut are associated with high fibre non-Western diets as they possess extensive repertoires of carbohydrate active enzymes that allow this species to metabolise complex polysaccharides. We thus hypothesised that other gut *Prevotella* species are also adapted to carbohydrate-related metabolism although their exact gene content likely differs compared with *P. copri*, thus allowing them to utilise distinct fractions of dietary fibre. To identify differences in gene content, we first predicted protein coding genes in each genome and then compared predicted genes to the KEGG Orthology (KO) database to infer metabolic potential. NMDS ordinations of KO counts indicated that genomes of prevalent and relatively abundant species belonging to the two copri and stercorea groups identified above significantly differed in their gene content (Fig. S1), and this could be attributed to several pectin and hemicellulolytic enzymes detected in the copri group but missing from stercorea (Table S6). Hemicellulose is a polysaccharide polymer in plant cell walls and typically consists of sugars such as xylose, arabinose, mannose, galactose and rhamnose. The stercorea group either lacks or has fewer copies of genes such as xylanase (K01181), xylosidase (K01198), xylose symporters (K08138), mannosidase (K01218), pectinesterase (K01051), rhamnosidase (K05989) and endoglucanase (K01179) suggesting that stercorea species have reduced ability to utilise hemicelluloses compared with copri-related species. Instead, stercorea species may participate in breaking down oligosaccharides and glycoproteins based on detection of genes such as N-acetylglucosamine-6-phosphate deacetylase (K01443) and endo-beta-N-acetylglucosaminidase (K01227). In addition, stercorea species possess components of the glycine cleavage system and histidine utilisation system (hut) which could indicate a more extensive amino acid metabolism compared with the copri group. These carbohydrate utilisation patterns are also reflected in CAZy profiles indicating the stercorea group as non-hemicellulose degraders. CAZy families involved in hemicellulose degradation such as CE8 (pectin methylesterase), GH10 (β-1,4-xylanase), GH28 (polygalacturonase), GH30 (endo-xylanase), GH43_1 (β-xylosidase, a-l-arabinofuranosidase), GH67 (α-glucuronidase), GH115 (xylan α-1,2-glucuronidase) and GH141 (α-l-fucosidase, xylanase) were absent in nearly all stercorea group genomes, whereas the presence of CE3 (acetyl xylan esterase) and CE9 (*N*-acetylglucosamine 6-phosphate deacetylase) suggest a possible role in assisting with removal of ester modifications to facilitate carbohydrate degradation^52^ by *P. copri* or other gut bacteria with the necessary gene repertoire (Table S7) (Fig. 3). The presence of GH33 (sialidases) and GH123 (N-acetylgalactosaminidases) further suggest that the stercorea species could metabolise sialic acids derived from dietary (e.g. glycolylneuraminic acid in red meat^53^) and/or human glycans (e.g. acetylneuraminic acid) and are likely to utilise these substrates in addition to or instead of dietary fibre. Other *Prevotella* species such as sp001275135 prevalent in France and Spain possess hemicellulolytic CAZy families akin to copri, whereas sp002299635 and sp003447235 are more similar with stercorea in that they lack hemicellulolytic families. These similarities in CAZy content among species are supported by an NMDS of total CAZy counts showing two broad types of CAZy profiles in human gut *Prevotella*, one representing the copri group and the other the stercorea group with reduced capacity to utilise hemicelluloses (Fig. S2).

To examine whether diet may influence the distribution of gut *Prevotella* species, we assessed dietary intake information collected as part of the Yunnan gut microbiome study^25^ together with abundance values obtained from metagenome read mapping (see section above). The Yunnan survey was comprised of 881 individuals representing six ethnic groups in Yunnan province; 427 of the 881 were recruited from rural counties and another 454 from urban Kunming (capital of Yunnan province). A total of 676 individuals provided their past month dietary intake through a binary response questionnaire. We first established that diet primarily differed among the ethnic groups followed by rural/urban status (p < 0.05, PERMANOVA) (Table S8). While the Zang (Tibetan) diet was most distinct compared with other ethnic groups (Fig. S3A), total *Prevotella* relative abundance in the Zang was comparable to several ethnicities (11.7% vs. 10.9–15.5%) whereas the Dai (8.0%) and Han (3.9%) had significantly lower relative abundances (Fig. S3B). Across all ethnicities, only coffee consumption was associated with an increased *Prevotella* relative abundance (p < 0.05, generalised linear model with FDR adjustment) (Table S9) although coffee consumption was reported in only 19 individuals. Other foods in the questionnaire were not correlated with either total *Prevotella* relative abundance or with abundances of the two stercorea and copri lineages identified above. Similarly, the low relative abundances of *Prevotella* in the Dai and Han could not be attributed to any specific foods included in this questionnaire. These observations indicate that non-dietary influences (e.g. genetics, lifestyle) could play a role in gut *Prevotella* associations in the Yunnan cohort, although more in-depth and quantitative dietary information in addition to existing data provided in that Yunnan study may be required to resolve exact correlations between these factors and diet.

## Discussion

The *Prevotella* genus is a lineage of interest as its member species are a common feature of the human gut microbiome and have been repeatedly implicated in health and disease. *P. copri* is often the primary focus of the *Prevotella* genus, but there is increasing incentive to catalogue and characterise other gut *Prevotella* species. For example, a gut microbiome survey of competitive cyclists revealed enrichment of *P. copri* and several unidentified *Prevotella* species correlated with exercise duration^21^. Another study of gut microbiome development in malnourished children reported that children who received a food formula designed to stimulate development of a healthy gut microbiota^54^ showed greater enrichment of *P. copri* and other unnamed *Prevotella* species compared with existing nutrition regimes^22^. To capture gut *Prevotella* diversity without relying on known marker genes (e.g. MetaPhlAn) or genomes deposited in RefSeq (Kraken2), our workflow in this study classifies metagenome reads against a large collection of human gut-derived *Prevotella* MAGs to better resolve and estimate abundances of *Prevotella* species in the human gut^15,17,18^.

We show that the most numerically abundant *Prevotella* species in the human gut are largely comprised of two distinct lineages, one closely related to and including *P. copri* while the other to *P. stercorea*. Both the copri and stercorea groups were more relatively abundant in rural non-Westernised human populations, suggesting that the presence of stercorea-related lineages is also driven by diet and lifestyle factors akin to *P. copri*^2–4^. Using the *P. copri* phylogenetic framework inferred by Tett et al.^1^, the copri group of species we identified here largely corresponded with the four clades circumscribed in that study and the potential for carbohydrate metabolism detected in this group also broadly mirrors that reported by Tett et al.^1^. While *P. copri* are known hemicellulose degraders^55,56^, compared with *P. copri* the stercorea group occupies a separate functional niche categorised by a lack of hemicellulolytic gene families. In addition, the stercorea group possesses carbohydrate esterase families that were comparatively much less common in the copri group. From these features, we posit that a possible role of the stercorea group is to remove ester-based modifications from dietary carbohydrates to facilitate hydrolysis by gut species with the necessary hydrolytic capacity^52^. Additionally, the stercorea group lineages possess sialidases. Sialic acids have been demonstrated to play roles in processes such as mediating interaction between immune cells, pathogen binding to human cells^57^ and inflammation in the gut^58^. The presence of sialidases in the stercorea group therefore suggests that stercorea-related bacteria have important functions that could impact human health.

The main limitation of this work is the lack of detailed lifestyle and dietary information needed to investigate whether distribution of the several gut *Prevotella* identified here is influenced by lifestyle and/or diet. Existing evidence indicate that *P. copri* is associated with dietary fibre intake^59^, but we did not observe such associations with the limited dietary data (yes or no responses) provided in the Yunnan study^25^. In addition, our metabolic inferences of the various *Prevotella* species are based on genome sequences and thus need to be validated by isolation and laboratory culture experiments. Nevertheless, since low fibre diets can result in poor gastrointestinal health^60^ and *Prevotella* are one of the major fibrolytic bacteria in human guts^61^, we foresee that there will be increased interest in exploiting *Prevotella* to potentially improve human health and nutrition. Our findings of distinct *Prevotella species* in the human population underscores the need to classify the human gut microbiome in greater resolution to understand and exploit the metabolic potential of gut microorganisms.

## Supplementary Information

Supplementary Information.

Supplementary Tables.

## Data Availability

Metagenome-assembled genomes used in this study can be downloaded from https://drive.google.com/file/d/1hLaPiR1qHKBeDsK0A3WLzk3ADgCFkrNH/view?usp=sharing. Codes used to generate genomes are provided in supplementary file 1.
