# Supplementary material for: Prevotella species in the human gut is primarily comprised of Prevotella copri, Prevotella stercorea and related lineages
Source: Sci Rep. 2022 May 31;12:9055. doi: 10.1038/s41598-022-12721-4 (PMC9156738; doi:10.1038/s41598-022-12721-4)
Supplement: Supplementary file 1 — Supplementary Information. [file 41598_2022_12721_MOESM1_ESM.pdf]

Prevotella species in the human gut is primarily comprised of _Prevotella copri, Prevotella stercorea_ and related lineages

Yun Kit Yeoh, Yang Sun, Lawrence Yuk Ting Ip, Lan Wang, Francis KL Chan, Yinglei Miao, Siew C Ng

Supplementary figures S1-S3

Supplementary file 1

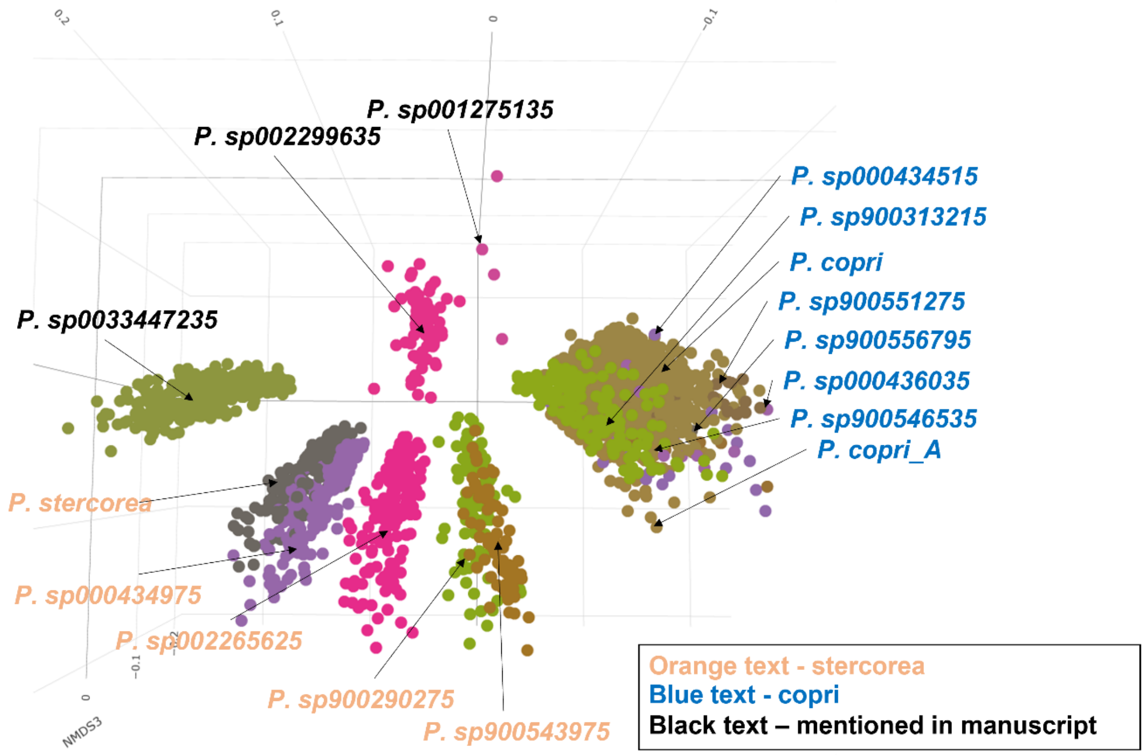

Figure S1. Nonmetric multidimensional scaling (NMDS) ordination of Kyoto Encyclopedia of Genes and Genomes (KEGG) Orthology (KO) counts identified in human gut *Prevotella* genomes. Taxonomy labels are coloured to indicate copri- (blue) and stercorea-related lineages (orange) as well as others mentioned in the manuscript (black).

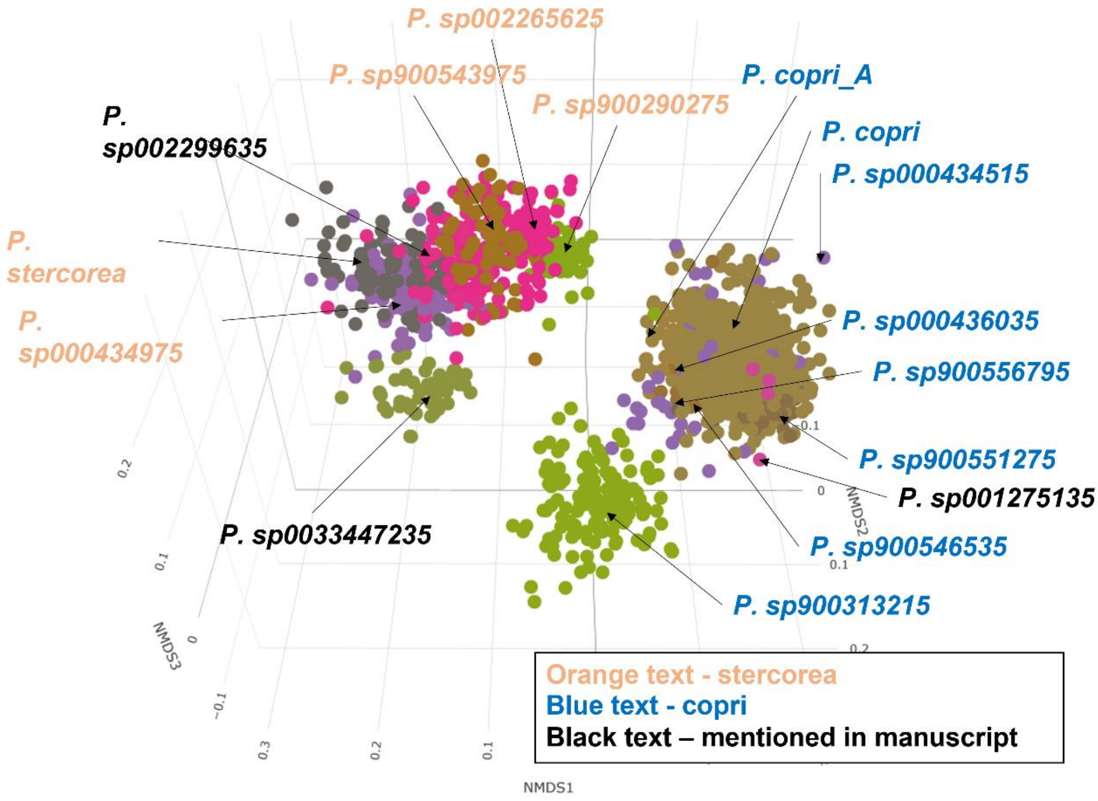

Figure S2. Nonmetric multidimensional scaling (NMDS) ordination of carbohydrate active enzyme (CAZy) counts identified in human gut *Prevotella* genomes. Taxonomy labels are coloured to indicate copri- (blue) and stercorea-related lineages (orange) as well as others mentioned in the manuscript (black).

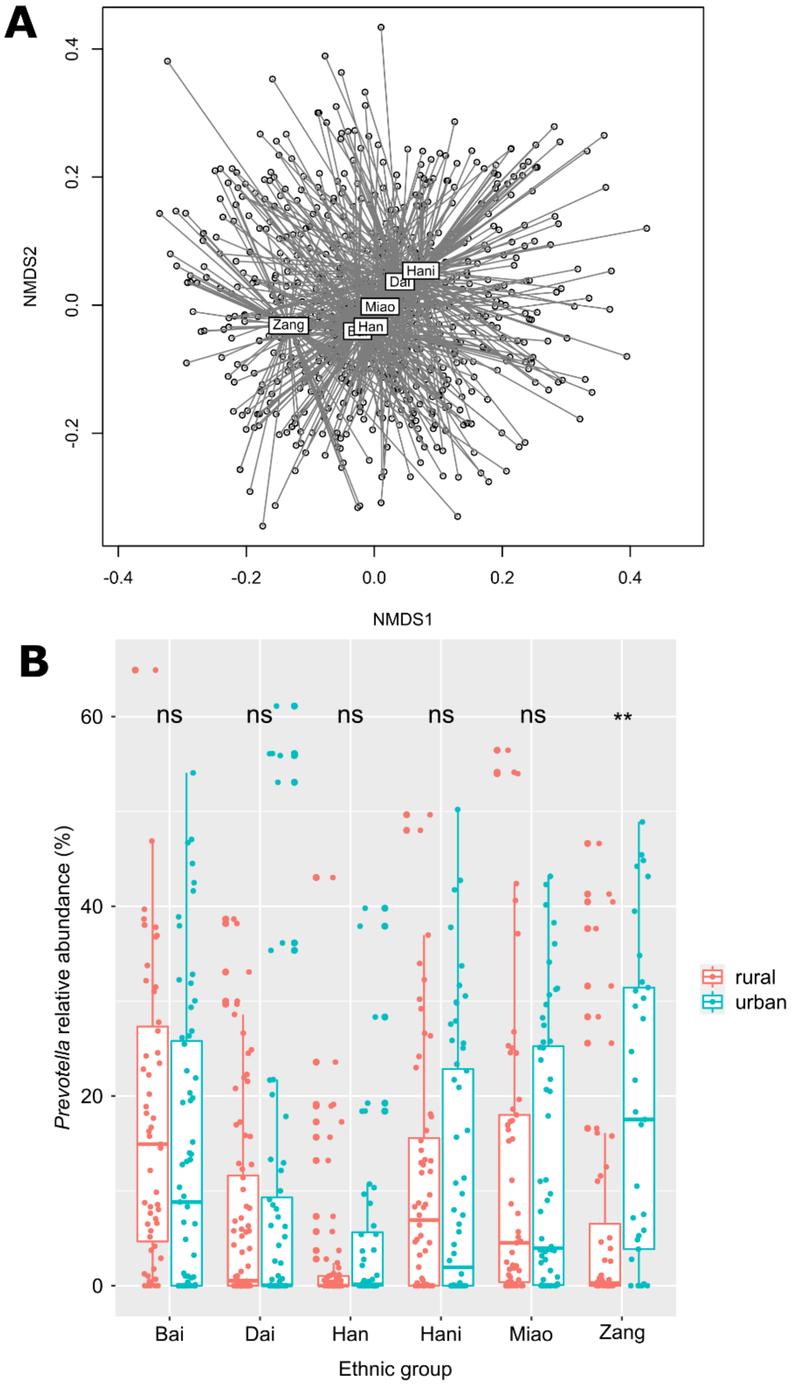

Figure S3. (A). Nonmetric multidimensional scaling (NMDS) ordination of self-reported dietary preferences among ethnic groups in the Yunnan cohort. Dietary data was obtained from Sun et al., 2021. (**B**). Box and whisker plot of *Prevotella* relative abundances in the Yunnan cohort. The bottom, middle and top borders of the boxes represent the first, median, and third interquartile ranges, respectively. Whiskers extend to 1.5x interquartile range. Only the Zang group had significantly distinct Prevotella relative abundance when comparing between rural and urban Zang individuals ($p < 0.001$, Kruskal-Wallis test).

Supplementary file 1

Genome binning for Hong Kong and Yunnan gut metagenomes

0. Create a batchfile

List all sample IDs in a file, one per line. This is the batchfile. The commands below assume metagenome read files are named sample01_1.fastq.gz and sample01_2.fastq.gz, sample IDs being sample01, sample02, … and so on.

1. Quality filtering metagenome reads using Trimmomatic

```
cat batchfile | parallel -j3 "java -jar
/srv/sw/trimmomatic/0.39/trimmomatic-0.39.jar PE -threads 4
{}_1.fastq.gz {}_2.fastq.gz {}_trimmed_1.fastq.gz
{}_trimmed_unpaired_1.fastq.gz {}_trimmed_2.fastq.gz
{}_trimmed_unpaired_2.fastq.gz
ILLUMINACLIP:/srv/sw/trimmomatic/0.39/adapters/TruSeq3-PE-
2.fa:2:30:10 LEADING:3 TRAILING:3 SLIDINGWINDOW:4:15
CROP:10000 HEADCROP:0 MINLEN:50"
```

2. De novo assembly using Megahit

```
cat batchfile | parallel -j1 "megahit -t 12 -m 0.7 -1
{}_trimmed_1.fastq.gz -2 {}_trimmed_2.fastq.gz -r
{}_trimmed.fastq.gz -o denovoassemblies/{} --out-prefix {}"
```

3. Read mapping using BWA

```
cat batchfile | parallel -j1 "bwa mem -t12
denovoassemblies/{}/{}.contigs.fa {}_trimmed_1.fastq.gz
{}_trimmed_2.fastq.gz | samtools view -Subh - | samtools sort
-m 5G -@ 4 -o {}.bam -"
```

4. Genome binning using Metabat and Maxbin

```
cat batchfile | parallel -j4 "jgi_summarize_bam_contig_depths
--outputDepth depth_{}.txt {}.bam"
```

4.1 Metabat v2.10.2

```
cat batchfile | parallel -j1 "metabat -i
denovoassemblies/{}/{}.contigs.fa -a depth_{}.txt -o
pop_genome_binning/metabat2102/{} -t 12"
```

4.2 Metabat v2.12.1

```
cat batchfile | parallel -j1 "metabat2 -i
denovoassemblies/{}/{}.contigs.fa -a depth_{}.txt -o
pop_genome_binning/metabat2121/{} -t 12"
```

4.3 Maxbin

```
cat batchfile | parallel -j2 "cut -f1,3 depth_{}.txt | sed
'1d' > maxbin_abund_{}

run_MaxBin.pl -contig denovoassemblies/{}/{}.contigs.fa -abund
maxbin_abund_{} -out pop_genome_binning/maxbin/{} -thread 6"
```

5. Merging genome bins using DAS Tool

Create a "scaffold to bin" mapping file from metabat output:

```
for i in `find pop_genome_binning/metabat2102/*/ -maxdepth 0`;
do cd $i; b=$(cut -f3 -d"/" <(echo $i)); for a in *.fna; do
grep ">" $a | sed "s/$/\t$a/" | sed 's/>//' | sed 's/.fna//';
done > pop_genome_binning/dastool/metabat_scaff2bin_$b; done

for i in `find pop_genome_binning/metabat2121/*/ -maxdepth 0`;
do cd $i; b=$(cut -f3 -d"/" <(echo $i)); for a in *.fna; do
grep ">" $a | sed "s/$/\t$a/" | sed 's/>//' | sed 's/.fna//';
done > pop_genome_binning/dastool/metabat2_scaff2bin_$b; done
```

Create a “scaffold to bin” mapping file from maxbin output:

```
for i in `find pop_genome_binning/maxbin/*/ -maxdepth 0`; do
cd $i; b=$(cut -f3 -d"/" <(echo $i)); for a in *.fasta; do
grep ">" $a | sed "s/$/\t$a/" | sed 's/>//' | sed
's/.fasta//'; done >
pop_genome_binning/dastool/maxbin_scaff2bin_$b; done

cat batchfile | parallel -j1 "DAS_Tool -i
metabat_scaff2bin_{},metabat2_scaff2bin_{},maxbin_scaff2bin_{}
-c denovoassemblies/{}/{}.contigs.fa -o
pop_genome_binning/dastool/{} -l metabat,metabat2,maxbin –
threads 5 –write_bins 1 –write_bin_evals 1"
```

Check quality and infer taxonomy for all genomes

6. Check genome quality using checkM

first put all genomes created by DASTool and downloaded genomes in a directory called ‘checkm_in/’

```
checkm lineage_wf -x fa -t 32 –pplacer_threads 30 checkm_in/
checkm_out/
```

7. Infer genome phylogeny using GTDB-Tk

```
gtdbtk classify_wf –genome_dir checkm_in/ --out_dir gtdbtk_out
-x fa –cpus 12
```

8. Run Kraken2 on genomes

*.fa refers to all genomes in the current directory e.g. when the below command is run in checkm_in/

```
find *.fa | sed 's/.fa//' | parallel -j3 "kraken2 –db
k2_pluspf_20210517 –threads 8 –output - --report
kraken2/report_{}.txt {}.fa"
```

9. Dereplicate genomes using CoverM

Directory quality_genomes/ contains genomes that pass quality filter

```
coverm genome –genome-fasta-directory quality_genomes/ -x fa –
dereplicate –dereplication-ani 99 –threads 12 –dereplication-
precluster-method finch –dereplication-output-representative-
fasta-directory dereplicated_99/
```

```
coverm genome –genome-fasta-directory quality_genomes/ -x fa –
dereplicate –dereplication-ani 95 –threads 12 –dereplication-
precluster-method finch –dereplication-output-representative-
fasta-directory dereplicated_95/
```

10. Infer phylogenetic tree using IQ-TREE2

```
iqtree2 -s gtdbtk_out/gtdbtk.bac120.fasta –seed 1234 -T 12 -m
MFP -B 1000 -alrt 1000
```

11. Relative abundance estimates using CoverM

replace sample01, sample02, … in the command below with actual sample IDs

```
coverm genome --coupled <sample01_trimmed_R1.fastq.gz
sample01_trimmed_R2.fastq.gz sample02_trimmed_R1.fastq.gz
```

```
sample02_trimmed_R2.fastq.gz ... > --genome-fasta-directory
dereplicated_95/ --threads 12 --methods relative_abundance -o
relabun.tsv
```

12. Annotating genomes using Prokka and Panaroo

```
find *.fa | sed 's/.fa//' | parallel -j2 "prokka --outdir
prokka_annotation/{} --prefix {} --centre X --compliant --cpus
4 {}.fa"
```

link all gff files created by Prokka into a single directory for panaroo

```
panaroo -i *.gff -o panaroo_results --clean-mode strict
```

13. Identifying KEGG orthology (KO) carbohydrate active enzymes (CAZy) using EnrichM

```
enrichm annotate --output enrichm_annotate_ko/ --
genome_directory dereplicated_99/ --ko --threads 12

enrichm annotate --output enrichm_annotate_cazy/ --
genome_directory dereplicated_99/ --cazy --threads 12

enrichm classify --output enrichm_classify/ --
genome_and_annotation_matrix enrichm_annotate_ko/
frequency_table.tsv

enrichm classify --output enrichm_classify/ --
genome_and_annotation_matrix enrichm_annotate_cazy/
frequency_table.tsv
```
